# LGBTQ+ health research guides at North American health sciences libraries: a survey and content analysis

**DOI:** 10.5195/jmla.2021.1189

**Published:** 2021-07-01

**Authors:** Gregg A. Stevens, Francisco J. Fajardo

**Affiliations:** 1 gregg.stevens@stonybrook.edu, Senior Assistant Librarian/Health Sciences Librarian, Liaison to the School of Nursing, Stony Brook University, Stony Brook, NY; 2 francisco.fajardo@fiu.edu, Information & Instruction Services Librarian, Medical Library, Herbert Wertheim College of Medicine, Florida International University, Miami, FL

**Keywords:** LGBTQ+, health information, research guides, health disparities, health sciences libraries, transgender, HIV/AIDS, women's health

## Abstract

**Objectives:**

Current literature recommends online research guides as an easy and effective tool to promote LGBTQ+ health information to both health care providers and the public. This cross-sectional study was designed to determine how extensive LGBTQ+ health guides are among hospital and academic libraries and which features are most prevalent.

**Methods:**

In order to locate LGBTQ+ health guides for content analysis, we searched for guides on the websites of libraries belonging to the Association of Academic Health Sciences Libraries (AAHSL) and the Canadian Association of Research Libraries (CARL). Additionally, we searched the Springshare interface for LibGuides with the word “health” and either “LGBT” or “transgender.” Content analysis was performed to identify major characteristics of the located guides, including target audience and the information type provided.

**Results:**

LGBTQ+ research guides were identified for 74 libraries. Of these, 5 were hospital libraries, and the rest were academic libraries. Of 158 AAHSL member libraries, 48 (30.4%) had LGBTQ+ guides on their websites. Nearly all guides (95.9%) provided general LGBTQ+ health information, and a large majority (87.8%) also had information resources for transgender health. Smaller percentages of guides contained information on HIV/AIDS (48.6%) and women's health (16.2%).

**Conclusions:**

Even though literature recommends creating LGBTQ+ health guides, most health sciences libraries are missing an opportunity by not developing and maintaining these guides. Further research may be needed to determine the usage and usefulness of existing guides and to better identify barriers preventing libraries from creating guides.

## INTRODUCTION

The lesbian, gay, bisexual, transgender, queer, and plus (LGBTQ+) community has a long-recorded history of facing discrimination, social stigma, and issues leading to health disparities. Often classified as a sexual and gender minorities subgroup, research shows that members of the LGBTQ+ community are more likely to have mental health disorders, adverse childhood experiences, increased alcohol/drug use, and an increased risk of sexually transmitted infections (STIs) [1–3]. Beyond these disparities, members of this community also have higher rates of smoking, stroke, and obesity than their heterosexual counterparts [4, 5]. These health disparities put many, especially LGBTQ+ youth, at a greater risk of suicide, bullying, and homelessness [6].

While the LGBTQ+ community overall faces many difficulties, these stigmas and health disparities are doubled for transgender members of the community due to a lack of legal protections [6, 7]. Transgender persons are at higher risk for alcohol/drug use, STIs, and violence compared with cisgender (birth sex consistent with sexual/gender identity) persons. Transgender women have a particularly high risk of HIV infection, with an odds of infection 34.2 times higher than that of the overall US population [8–10]. Among transgender women of color (including African American and Latina transgender women), the rate of HIV prevalence is almost double that of the general population due to commercial sex work and drug use [11]. Despite advances in prevention strategies and the use of pre-exposure prophylaxis (PrEP) and post-exposure prophylaxis (PEP) medications, HIV infection remains an issue for this segment of the community.

HIV infection remains a health disparity not only for transgender women but also for men who have sex with men (MSM). MSM is a category which encompasses gay men and bisexual men as well as men who may not identify as gay or bisexual but who still engage in sexual activity with men. In 2018, gay and bisexual men represented 69% of new HIV infections despite ongoing HIV prevention efforts through education and PrEP availability [12]. MSM are also at greater risk for contracting other STIs. Of particular note is the high prevalence of syphilis infection, with MSM comprising the majority of new syphilis diagnoses in the United States [13, 14].

Cisgender individuals within the LGBTQ+ community similarly face a number of health disparities in comparison with the general population. Research shows that lesbians and bisexual women, as compared with heterosexual women, are more likely to be overweight or obese [15]. They are also more likely to drink, smoke, or use drugs, which, along with increased body weight, puts LGBTQ+ women at an elevated risk for other conditions such as cardiovascular disease, certain types of cancer, and type 2 diabetes [15, 16].

In part, members of the LGBTQ+ community continue to face these disparities due to the continued discrimination and lack of training of health care professionals. Culturally sensitive physicians, medical students, and other health care providers are needed to prevent many of these barriers to health care. For example, LGBTQ+ patients often do not disclose their experiences to health care providers [17]. More training, education, and resources for these providers are needed to prevent patients from experiencing discriminatory medical care.

A review of the library sciences literature reveals a number of articles that discuss guides and other online resources as a way for libraries to reach out to the LGBTQ+ community and provide access to information, including information on health topics. Online library resources for LGBTQ+ information needs have an important role in higher education. Mehra and Braquet (2011) describe the use of an LGBT research guide at a major university library and recommend improved access to LGBTQ information online as a method for university reference librarians to be more engaged with students during the coming-out process [18]. In his study of academic libraries and campus climate for LGBT students, Ciszek (2011) noted that guides focused on LGBT content can provide one means of creating a welcoming environment. However, he noted that only 25% of the academic libraries in his survey actually had any sort of online guide for LGBT students or highlighting LGBT resources [19]. Lupien (2007), in his study of library usage by students in GLBT and Sexual Diversity studies programs, found that students believed libraries could do more to promote their resources. One of the common suggestions from respondents was that libraries should create pages online to highlight their LGBT resources [20]. Stewart and Kendrick (2019) recommended the use of research guides to promote library LGBTQ+ resources, as well as resources available to students on campus and in the local community [21]. In yet another study, LGBTQ students expressed an interest in subject guides devoted to LGBTQ research areas [22]. While the guides in these studies were not specific to health, they illustrate that guides can be useful and effective tools to provide information to the LGBTQ+ community. Additionally, guides specific to a campus, such as those mentioned above, provide the value of highlighting local resources, which can be harder to find than widely known resources.

In their article on providing health information to the LGBTQ+ community, Hawkins et al. (2017) note that online information resources such as research guides, as well as physical materials in the library itself, can be a means for librarians to disseminate quality consumer health information to the LGBTQ community [23]. Other studies have shown how members of the LGBTQ+ community have sought out health information online. Grov et al. (2014) provide a historical review of how gay and bisexual men have used the internet since the 1990s for obtaining health information, focusing on sexual health information [24]. Lindley (2012) looked at consumer health websites for sexual health information targeted at lesbians. The lack of appropriate, well-written health information in this study shows that there is a lack of targeted health information on certain topics for a major component of the LGBTQ+ community [25].

Morris and Roberto (2016) suggest that library guides or resource lists on LGBTQ health could be helpful in meeting the information needs of LGBTQ health professionals. They stated that these resources could indicate the subject expertise of the librarian behind them, as well as the availability of the librarian to partner with health care professionals [26]. Hawkins et al. (2017) recommend several ways in which medical librarians can provide consumer health information to improve LGBTQ health, including the creation of research guides. They also recommended the use of LibGuides for health care providers, so that they would have easy access to quality LGBTQ health information [23]. In their profile of health sciences librarian outreach to the LGBTQ+ community, Stevens et al. (2019) build on the Hawkins article by giving specific examples of how online research guides and portals have been used effectively to provide access to LGBTQ+ health information. For example, they discussed how a librarian at Augusta University in Georgia established an information portal that provided information on LGBTQ+ health topics to both patients at the university's Equality Clinic as well as to its providers. Additionally, they describe how a librarian at Dalhousie University in Nova Scotia partnered with the provincial health authority and local public libraries to establish a research guide to provide LGBTQ+ health information to three audiences: researchers, health care providers, and the general public [27]. These two case studies are examples of online guides and resources being used successfully to provide quality LGBTQ+ health information.

This previous research highlights the benefits of online resources such as subject guides for LGBTQ+ topics, as well as desire among potential users for these guides. However, a number of these LGBTQ+ guides lack health information addressing the specific needs of the community, which does nothing to help alleviate the need for health information and by extension potentially reduce LGBTQ+ health disparities. LGBTQ+ health guides are by no means universally found on the websites of health sciences libraries in North America, despite the recommendations of numerous authors. The objective of this cross-sectional study was to assess the degree to which academic and health sciences librarians are creating and maintaining LGBTQ+ health LibGuides at US and Canadian colleges and universities.

## METHODS

In order to locate as many LGBTQ+ health science research guides as possible, we used a three-part methodology. All of the searches and analyses were conducted in July 2019.

First, the website for the Association of Academic Health Sciences Libraries (AAHSL) was accessed, and the organization's membership directory was consulted to provide a standard list of health sciences libraries in the United States [28]. The library websites for 158 US member libraries were accessed, and results were recorded in a Google Sheets spreadsheet to allow for easy collaboration between researchers. Upon identification of a library's research guides on its website, the list of guides was scanned for any guides which obviously offered LGBTQ+ health content. If no guide was easily identified, then the search box was used to search for “LGBT” or “transgender.” If the search returned any results, the located pages were examined to determine if they were entirely devoted to LGBTQ+ health content.

AAHSL membership is open to libraries serving medical schools in the United States and Canada that are affiliated with the Association of American Medical Colleges. However, because there were only seven Canadian members of AAHSL at the time of this study, the decision was made that another method should be chosen to identify possible Canadian libraries. There is no equivalent organization to AAHSL in Canada. As a substitute, we used the member list of the Canadian Association of Research Libraries (CARL). In 2018, there were twenty-nine CARL member libraries [29]. Of the seven Canadian AAHSL members, all but one was also a CARL member. The search protocol used for the AAHSL member websites was also used for the twenty-nine CARL member websites. Results were recorded on a separate tab in the Google Sheets spreadsheet.

Finally, to locate other potential academic libraries with LGBTQ+ health guides but were not members of either AAHSL or CARL, a search was conducted on the LibGuides Community portal [30]. The search terms “LGBTQ health” and “transgender health” were used. Results were recorded on a third tab of the Google Sheets spreadsheet. For the purposes of this study, only results from hospital or academic libraries were included. A few guides were found from public and school libraries, but these were excluded.

Because the focus of this study was to find and assess guides to promote health information among health care professionals, health care students, or consumers, a few exclusion criteria were established. Guides were excluded from consideration if the guide or guide page did not focus exclusively on LGBTQ+ health. For example, a page with multiple categories of LGBTQ+ resources, with a box for health resources included, would be excluded because its primary focus was not health. As previously described, guides from public and school libraries were not included. Finally, guides created for specific courses were excluded because they have a specific target audience and are not designed for wider usage.

After identification of the guides, we performed content analysis to determine whether certain information categories were present or absent within the guides [31]. We focused on three basic themes in the analysis: the intended audience of the guides, the types of materials highlighted, and the categories of LGBTQ+ health information provided. The coding categories selected for the guides' target audiences were health care providers, health care students, and the general public. It was possible for a guide to be coded for more than one audience. These three audiences were chosen because they best represent the range of potential patrons who might access research guides from an academic health sciences library or hospital library. The material categories were books, databases, and websites, as these three areas cover most text-based health information materials. The four categories of LGBTQ+ health selected for coding were general LGBTQ+ health information, transgender health information, HIV/AIDS information, and information on women's health. These four categories were chosen because of their relevance to major health disparities in the community as described in recent literature on LGBTQ+ health and librarianship [23, 27, 32]. Coding was conducted in a Google Sheets spreadsheet.

## RESULTS

LGBTQ+ health research guides were located for a total of 74 institutions (Appendix A). Five of the institutions were hospital libraries, and the remainder were academic libraries. From the search of the 158 AAHSL member libraries, we found 48 (30.4%) libraries with LGBTQ+ health guides. Six out of the 29 member libraries of CARL had LGBTQ+ health guides (20.7%). Three libraries with these guides were included in both the AAHSL and CARL cohorts: McGill University, McMaster University, and the University of Manitoba. The guides were fairly evenly geographically distributed, with 25% of the guides from libraries in the US Northeast, 29% from the Midwest, 21% from the West, and 16% from the South. The remaining 8% were the six Canadian libraries.

Regarding the guides' target audiences, just over one-third of the guides (n=28, 37.8%) featured consumer health information targeted to the general public. Higher percentages of guides appeared to target health care professionals (n=42, 56.8%) and university students (n=52, 70.2%), which may be expected considering that the publishing libraries were either academic or hospital libraries.

Nearly all of the guides (n=71, 95.9%) featured general LGBTQ+ health information, with no particular focus other than the health of the LGBTQ+ community broadly. Most of the guides (n=65, 87.8%) had information specific to transgender health. Just under half of the guides (n=36, 48.6%) featured HIV/AIDS information, and an even smaller number (n=12, 16.2%) contained information specific to women's health. All but one of the guides featured links to websites (n=73, or 98.6%). Nearly two-thirds of guides (n=54, 73.0%) provided book information, and just under half (n=36, 48.6%) had links to library databases.

## DISCUSSION

Content analysis of the information categories provides some insight into the health priorities of the LGBTQ+ community at present. As one might expect, general health information for the community was found on the vast majority of guides to appeal to the widest number of information seekers. The high percentage of guides featuring transgender health (nearly 88%) is encouraging, as it shows that awareness of the transgender members of the community and their specific health needs is more widespread than it might have been in the past.

More surprising, perhaps, was how few guides featured specific information on two long-time core areas of LGBTQ+ health: women's health and HIV/AIDS. Since the 1980s, HIV/AIDS has dominated discussions of health for the community. Libraries and librarians played a role in providing information on HIV/AIDS to the community during the early decades of the disease, as is reflected in AIDS reference books by librarians from the early 1990s like *AIDS Information Sourcebook* and *HIV/AIDS Internet Information Sources and Resources* [33, 34]. However, the dramatic impact of HIV on those with the virus is no longer as severe as it once was. Drugs are widely available that can reduce the viral load in HIV+ individuals to levels where the virus is undetectable and therefore untransmissible. These individuals are living much longer lives, so the widespread illnesses and deaths of the 1980s and 1990s have become uncommon. Other drugs are readily available that prevent transmission of the HIV virus to HIV-negative individuals. The most commonly available drug for PrEP, Truvada, became available as a generic drug in fall 2020 [35]. A second medication for PrEP in use, Descovy, was approved by the U.S. Food and Drug Administration and is believed to have fewer side effects [36]. Despite the wider availability of these drugs, usage remains low among MSM. According to the U.S. Centers for Disease Control and Prevention, only 21% of Hispanic/Latino and 19% of Black/African American MSM use some form of PrEP, while 31% of white MSM use PrEP [12]. Because medications to prevent and treat HIV infection and the ways to obtain the medications are always changing, quality HIV information in a library's LGBTQ+ health guide can provide objective information for both providers and the public.

An example of such a guide is the one created by librarians at the Florida International University (FIU) Herbert Wertheim College of Medicine Medical Library. Librarians, in collaboration with researchers from the medical school and school of public health, created a LibGuide on transgender resources based on feedback from a study examining disparities of transgender women of color in South Florida [11]. This study sought to identify the awareness and willingness to use PrEP among transgender women. The guide was designed to provide local, regional, and national health resources for health care providers and members of the South Florida transgender community. Resources included information on obtaining PrEP and contact information of medical providers specializing in transgender health, such as connecting patients to hormone replacement therapy, plastic surgeons specializing in gender-affirming surgery, and mental health professionals working with transgender patients. This guide was later used by the different local organizations working with this population as a reference for linkages to providers.

Because the majority of LGBTQ+ people with HIV/AIDS in the early days of the disease were men, the specific health needs of lesbian and bisexual women were often overlooked. However, there are a number of specific health disparities affecting these women, both in regard to increased risk of certain diseases and in decreased usage of preventative care such as mammography and Pap smears [37]. Considering that these disparities exist, the lack of specific resources for cisgender LGBTQ+ women in the research guides was surprising. As with HIV/AIDS information, there is perhaps a need for guides to highlight these resources more effectively for both providers and for consumers. One particularly good example of a guide with health information for lesbians is found on the website for the University of Michigan. On a page dedicated to lesbian health, there are links to seven websites on lesbian health, as well as links to lesbian support groups in the state [38].

These guides vary not only in their subject areas but also in the types of materials presented. Some are fairly basic, consisting of links to relevant databases, books, and websites. A few guides contained PubMed search strings that were constructed to help the user search for the latest journal articles. Other guides have elements which educate the reader on LGBTQ+ health issues and needs. For example, the University of Arizona's guide for health care providers on working with LGBTQIA+ (lesbian, gay, bisexual, transgender, queer, intersex, asexual, and others) patients includes a tab on health care–specific recommendations. This page includes descriptions of how specific groups within the community may feel anxious about their interactions with health care providers and suggestions on how providers can communicate more effectively and positively with their patients [39].

Some guides highlighted national, international, and local organizations. Guides published by college and university libraries often connected users to student services organizations on campus, such as gay/straight alliances and medical professional associations. In this regard, the librarian is creating connections between the users and the greater community, which can lead to collaborations between librarians and the greater community. As described in the FIU study, librarians and public health faculty reached outside the university to work with local organizations to improve health knowledge and increase usage of available medical services [11]. The authors would argue that “making it local” could in fact be considered a recommended best practice in creating a guide for LGBTQ+ health. Partnering with organizations on campus and cosponsoring events are some ideas to maximize outreach, as providing the guide as a takeaway resource for these groups is a way to increase library visibility and improve the effectiveness of the other groups.

The presence or absence of a guide on a library website is most likely the result of the motivations of the librarian creating the guide. Stevens et al. (2019) hypothesize that outreach projects of health sciences librarians supporting the health of the LGBTQ+ community tend to be projects initiated by the librarians themselves rather than top-down initiatives within their libraries or larger institutions. These librarians could be considered early adopters of LGBTQ+ health initiatives and even activists for social justice by promoting health and social equity through access to health information [27]. The guides in this study could perhaps also be classified in the same manner, as many of the guides were most likely published by librarians who had a personal interest in promoting LGBTQ+ health. Librarians without a strong personal interest in LGBTQ+ health might not think to create a guide or might even have some discomfort in creating or supporting a guide with LGBTQ+ content [32]. However, it has been argued that the presence of a guide indicates to many patrons that the librarian behind the guide is an expert on the subject matter, which enhances the prestige of the librarian and, by extension, the library as a place to find quality information on LGBTQ+ health [26].

Just as the presence of these guides leads to speculation on the motivations of the guide creators, the absence of guides leads to a separate but related set of questions. A library without one of these guides does not necessarily indicate that the librarians or their institutions are uninterested in LGBTQ+ health or are antipathetic to the community. While Siegel et al. (2020) report a small number of homophobic librarians in their study, the vast majority of librarians displayed positive or neutral feelings toward the LGBTQ+ community [32]. Based on their study, it can be assumed that most librarians have good intentions regarding providing resources and services to LGBTQ+ patrons, so homophobia and transphobia are probably not demotivating factors for guide creation. Some libraries, for example, may have internal guidelines which dictate the content and style of their guides. These libraries may not allow guides that do not focus on a specific academic program or medical specialty, for example, so a guide on LGBTQ+ health would fall outside these standards and therefore not be permitted on a given library's website. Other libraries may choose not to create a guide because they do not perceive a need to create one, due to the existence of acceptable guides on other libraries' websites. This choice, though, gives the impression that the libraries either do not care strongly about the topic or that they do not consider themselves the best sources of information. It is our hope that, if librarians have not created guides because they consider other libraries' guides superior and numerous, the librarians should at least link to representative guides at other libraries so that their patrons could access quality LGBTQ+ health information. However, we believe that the most desirable response should be creation of their own guides. One of the most crucial elements of a guide focusing on consumer health is to link users to local resources, whether they be at the university or hospital that the library serves, or within the local geographic area. By making a guide local, it makes it more relevant to users.

While the health disparities that this study focused on are traditional core areas of LGBTQ+ health, other disparities may appear in the future and could be included in guides. At the time of writing of this article, the United States is dealing with the COVID-19 pandemic. There has been speculation that, as a result of some of the disparities mentioned above, such as smoking, obesity, hypertension, and HIV, the LGBTQ+ population is at an increased risk of the adverse effects of the coronavirus [40, 41]. The barriers to quality health care among many members of the LGBTQ+ community, especially those who are low-income, can also negatively impact their recovery from COVID-19 infection or can impact comorbid conditions [42, 43]. However, the literature on this topic is limited. With more research, the evidence linking COVID-19 outcomes to LGBTQ+ health disparities may become more evident. Nonetheless, COVID-19 health information tailored to the community could make for a useful addition to LGBTQ+ health guides.

## LIMITATIONS OF STUDY

Like many web pages, research guides are always in a state of flux. Our study only provides a snapshot based on an exploratory search in July 2019. It is possible that new guides have been created since this initial work or that guide content has changed. In fact, we hope that more guides have been created since the initial work, making the numbers reported here lower than they actually are at the time of this article's publication. It is also possible that, due to the difficulty in systematically searching Springshare's search engine, some relevant research guides were not retrieved by this method. Also, while the vast majority of libraries use Springshare's LibGuides platform for their research guides, there may be some smaller libraries which opt for another platform. Those libraries, if not members of AAHSL or CARL, would have been missed with the methodology used.

An additional limitation of this study was that only four health information categories were selected for coding. Further research could allow for inclusion of some areas not chosen for this basic initial analysis. Deeper coding to allow for subcategories could also be included, such as hormone therapy or mental health under the transgender health category, or PrEP and other HIV prevention measures under the HIV/AIDS category. Further deep coding could also allow for searching for information categories pertaining to specific LGBTQ+ communities. While this study did actively look for transgender health information, it could be possible to search for less recognized groups in the community, such as asexual or intersex.

## CONCLUSION

Research guides have been ubiquitous elements of library websites for decades. In academic libraries, these guides are often not used by students, for reasons including ignorance of the guides and a preference for Google searching first [44]. If one extrapolates these preferences and usage patterns to health care professionals and the public at large, then it would seem that creation and maintenance of guides is a low-impact librarian activity. However, health and medicine is an area in which the outcomes from using poor-quality information have much more serious ramifications. Patient outcomes are impacted from the quality of information located by physicians, nurses, and other health care workers. Health care workers should be motivated enough in their professional duties to seek out information even if it is not the most easily accessible. If the seeker of the health information is the patient themselves, or a friend or family member, the stakes are equally high. If a librarian can create a guide to assist in that information seeking, then they are potentially reducing barriers and indirectly improving health outcomes.

Despite the importance of locating quality LGBTQ+ health information, a major unanswered question is exactly how useful these guides are to the end users. Further research should look into how effective they are, perhaps with a cross-institutional study on usage numbers. No matter how often the guides are actually used, though, one could argue that the guides are important no matter the usage data. Considering that it is estimated that 4–5% of the US population identifies as LGBTQ+ [45], information sources focusing on the community's health will never see usage numbers as large as those for health information generally. Health information targeted to minority groups is important because they often have limited access to optimal health care and to resources that address many of the health disparities affecting them. Therefore, the focus should not be on guides with high impact, but rather on guides that promote health equity and practical recommendations of local resources and services.

## Data Availability

Data associated with this article are available through the Stony Brook University Academic Commons at https://commons.library.stonybrook.edu/universitylibra ries_data/1/.

## SUPPLEMENTAL FILES

**Appendix A:** Research guides included in content analysis
