# Supplementary material for: LGBTQ+ health research guides at North American health sciences libraries: a survey and content analysis
Source: J Med Libr Assoc. 2021 Jul 1;109(3):406–13. doi: 10.5195/jmla.2021.1189 (PMC8485968; doi:10.5195/jmla.2021.1189)
Supplement: Supplementary file 1 — Appendix A: Research guides included in content analysis [file jmla-109-3-406-s01.docx]

**Appendix A**

**Research Guides Included in Content Analysis**

| **Institution** | **URL** | **State/Province** |
| --- | --- | --- |
| Alabama College of Osteopathic Medicine | <https://libguides.acomedu.org/LGBTQ> | Alabama |
| Jacksonville State University | <https://libguides.jsu.edu/c.php?g=498565&p=3493445> | Alabama |
| University of Arizona | <https://libguides.library.arizona.edu/lgbtqresources/home> | Arizona |
| University of Arizona | <https://libguides.library.arizona.edu/LGBTQhealth> | Arizona |
| University of Arizona | <https://libguides.library.arizona.edu/LGBTQIAHealthcare> | Arizona |
| Northern Arizona University | <https://libraryguides.nau.edu/pbclibrary/lgbtdisparities>  <https://libraryguides.nau.edu/pbclibrary/transgenderhealthcare>  <https://libraryguides.nau.edu/LGBTQIAhealth>  <https://libraryguides.nau.edu/c.php?g=641059&p=4489500> | Arizona |
| AT Still University | <https://guides.atsu.edu/lgbtqihealth> | Arizona |
| Williams Institute/UCLA School of Law | <http://libguides.law.ucla.edu/c.php?g=183364&p=1208530> | California |
| Wilson Dental Library, University of Southern California | <https://wilson.usc.libguides.com/lgbtq> | California |
| Norris Medical Library, University of Southern California | <https://norris.usc.libguides.com/LGBTQhealth> | California |
| Northcentral University | <https://ncu.libguides.com/lgbtq> | California |
| University of California, San Francisco | <https://guides.ucsf.edu/lgbt_health> | California |
| University of Colorado HSL | <https://hslibraryguides.ucdenver.edu/c.php?g=259549&p=1732462> | Colorado |
| Greenwich Hospital Medical Library (CT) | <https://libguides.greenhosp.org/communityhealthsection/lgbtq> | Connecticut |
| Yale University | <https://guides.library.yale.edu/LGBTQHealth> | Connecticut |
| Goodwin College | <https://goodwin.libguides.com/c.php?g=29151&p=5367555> | Connecticut |
| Florida International University | <http://libguides.medlib.fiu.edu/transinfo> | Florida |
| Florida International University | <https://library.fiu.edu/FIULGBTQ/LocalHealthcare> | Florida |
| Palm Beach State College | <https://palmbeachstate.libguides.com/c.php?g=536664&p=4854247> | Florida |
| Valencia College | <https://libguides.valenciacollege.edu/c.php?g=513572&p=3526421> | Florida |
| Augusta University | <https://www.augusta.edu/library/greenblatt/lgbt/> | Georgia |
| University of Dubuque | <https://libguides.dbq.edu/LGBTQ/Health> | Iowa |
| Des Moines University | <https://lib.dmu.edu/su/culturalcompetency/lgbt> | Iowa |
| University of Illinois at Urbana-Champaign | <https://guides.library.illinois.edu/mbh/LGBTQIA> | Illinois |
| Midwestern University | <https://library.midwestern.edu/culture/lgbt> | Illinois |
| Indiana University School of Medicine-Indianapolis | <https://iupui.libguides.com/LGBTQ> | Indiana |
| Bethel College | <https://bethelks.libguides.com/c.php?g=730669&p=5218937> | Kansas |
| University of Kentucky | <https://libguides.uky.edu/c.php?g=555228&p=3815889> | Kentucky |
| Frontier Nursing University | <https://library.frontier.edu/LGBTQToolkit> | Kentucky |
| University of Louisville | <https://library.louisville.edu/kornhauser/competentcare> | Kentucky |
| University of Louisville | <https://library.louisville.edu/kornhauser/PBL/Y2C4/healthcare> | Kentucky |
| Tulane University | <https://libguides.tulane.edu/c.php?g=182435&p=1203799> | Louisiana |
| University of New Orleans | <https://libguides.uno.edu/c.php?g=150016&p=986415> | Louisiana |
| Harvard--Countway Library of Medicine | <https://guides.library.harvard.edu/lgbtqhealth> | Massachusetts |
| Baystate Health | <https://libraryinfo.bhs.org/lgbtq> | Massachusetts |
| Baystate Health | <https://libraryinfo.bhs.org/health_disparities/health_disparities_lgbtq> | Massachusetts |
| Baystate Health | <https://libraryinfo.bhs.org/c.php?g=785192> | Massachusetts |
| Massachusetts General Hospital | <https://libguides.massgeneral.org/lgbtqlibguide> | Massachusetts |
| Saginaw Valley State University | <https://librarysubjectguides.svsu.edu/c.php?g=597695&p=4138382> | Michigan |
| Henry Ford College | <https://hfcc.libguides.com/LGBTIQ/Health> | Michigan |
| Michigan State University | <https://libguides.lib.msu.edu/lgbthealth> | Michigan |
| Michigan State University | <https://libguides.lib.msu.edu/consumerhealth/lgbtq> | Michigan |
| University of Michigan | <https://guides.lib.umich.edu/lgbtqhealth> | Michigan |
| Mayo Clinic Library | <http://libraryguides.mayo.edu/glbt> | Minnesota |
| University of Minnesota | <https://libguides.umn.edu/transgender_topics> | Minnesota |
| Augsburg University | <https://library.augsburg.edu/lgbtqia/health> | Minnesota |
| Kansas City University of Medicine and Biosciences | <https://kcumb.libguides.com/lgbt> | Missouri |
| University of Missouri | <https://libraryguides.missouri.edu/c.php?g=28105&p=3558698> | Missouri |
| Washington University in St Louis | <https://beckerguides.wustl.edu/c.php?g=404453> | Missouri |
| Missouri Southern State University | <https://libguides.mssu.edu/c.php?g=423389&p=5771695> | Missouri |
| Northwest Missouri State University | <https://libguides.nwmissouri.edu/lgbtqia/health> | Missouri |
| University of North Carolina Chapel Hill | <https://guides.lib.unc.edu/lgbtqihealth> | North Carolina |
| University of North Dakota | <https://libguides.und.edu/c.php?g=91173&p=5445733> | North Dakota |
| Rowan University | <https://rowanmed.libguides.com/LGBTQAHealth> | New Jersey |
| William Paterson University | <https://guides.wpunj.edu/LGBT/LGBThealth> | New Jersey |
| Rider University | <https://guides.rider.edu/c.php?g=420661&p=2873897> | New Jersey |
| University of New Mexico Health Sciences Library | <https://libguides.health.unm.edu/lgbtq> | New Mexico |
| College of Southern Nevada | <https://libguides.csn.edu/c.php?g=525283&p=3616937> | Nevada |
| Albert Einstein College of Medicine | <https://libguides.einstein.yu.edu/lgbtq> | New York |
| St. John's University--Mental Health | <https://stjohnslis.libguides.com/c.php?g=895345&p=6441964> | New York |
| St. John's University--Sexual Health | <https://stjohnslis.libguides.com/c.php?g=895345&p=6441969> | New York |
| Pratt Institute | <https://libguides.pratt.edu/c.php?g=763492&p=5474985> | New York |
| Stony Brook University | <https://guides.library.stonybrook.edu/lgbtqhealth> | New York |
| SUNY Erie | <https://libguides.ecc.edu/c.php?g=881746&p=6335146> | New York |
| Westchester Community College (SUNY) | <https://researchguides.sunywcc.edu/c.php?g=751763&p=5455311> | New York |
| Icahn School of Medicine at Mount Sinai | <https://libguides.mssm.edu/specialtopics/lgbtqi> | New York |
| University of Toledo | <https://libguides.utoledo.edu/c.php?g=284199&p=1894862> | Ohio |
| Widener University | <https://widener.libguides.com/LGBT/web-resources> | Pennsylvania |
| Philadelphia College of Osteopathic Medicine | <https://libguides.pcom.edu/lgbt> | Pennsylvania |
| Brown University | <https://libguides.brown.edu/c.php?g=911447&p=6571192> | Rhode Island |
| University of South Dakota | <https://libguides.usd.edu/meded/LGBTIcare> | South Dakota |
| University of Tennessee-Knoxville | <https://libguides.utk.edu/transcultural/lgbtqia-persons> | Tennessee |
| Pellissippi State Community College | <https://lib.pstcc.edu/c.php?g=106838&p=693138> | Tennessee |
| Texas Tech University Health Sciences Center El Paso | <https://elpaso-ttuhsc.libguides.com/LGBTQ> | Texas |
| UT/MD Anderson Cancer Center | <https://mdandersontlc.libguides.com/lgbt> | Texas |
| University of North Texas | <https://guides.library.unt.edu/c.php?g=69966&p=452326> | Texas |
| James Madison University | <https://guides.lib.jmu.edu/LGBThealth> | Virginia |
|  |  |  |
| University of Manitoba | <https://libguides.lib.umanitoba.ca/lgbtq2health> | Manitoba |
| Dalhousie University | <https://dal.ca.libguides.com/LGBTQHealth> | Nova Scotia |
| McMaster University | <https://hslmcmaster.libguides.com/consumer/lgbtq> | Ontario |
| Queen's University | <https://guides.library.queensu.ca/lgbtq-health> | Ontario |
| McGill University | <http://libraryguides.mcgill.ca/lgbtqhealth> | Quebec |
| University of Saskatchewan | <https://libguides.usask.ca/c.php?g=706788> | Saskatchewan |
